# A Rare Form of Melanoma Masquerading as a Diabetic Foot Ulcer: A Case Report

**DOI:** 10.1155/2012/502806

**Published:** 2012-04-04

**Authors:** Susan Thomas, Yuan-Xiang Meng, Vijaykumar G. Patel, Gregory Strayhorn

**Affiliations:** ^1^Department of Family Medicine, Morehouse School of Medicine, 1513 East Cleveland Avenue, Building 100, Suite 300A, Atlanta, GA 30344, USA; ^2^Department of Surgery, Morehouse School of Medicine, 720 Westview Drive SW, Atlanta, GA 30310, USA

## Abstract

*Background*. Acral lentiginous melanoma (ALM) is a less-common form of melanoma in US, and it accounts for about 5% of all diagnosed melanomas in US. ALM is often overlooked until it is well advanced because of the lesion's location and its atypical appearance in the early stages. We present a case of ALM initially presented as a diabetic foot ulcer. *Case Report*. An 81-year-old man initially presented to the primary care clinic with a right foot diabetic ulcer. There was a large plantar, dark-colored ulcer that bled easy. Initial excision biopsy revealed Clark's Level IV ALM. Subsequent definitive wide excision and sentinel node biopsy confirmed ALM with metastasis to inguinal lymph nodes (stage IIIb). The treatment included wide margin excision of the lesion with en bloc amputations of 4th and 5th toes, followed by adjuvant chemotherapy. *Discussion*. The development of ALM may potentially relate to diabetes as a reported higher prevalence of diabetes with ALM patients. *Conclusion*. The difficulty in early diagnosing of ALM remains as a formidable challenge particularly in diabetic patients who commonly develop plantar foot ulcers due to the diabetic neuropathy. This case reiterates the importance of a thorough foot exam in such patients.

## 1. Introduction

Diabetic patients have been estimated to have a lifetime risk of 15% of developing a neuropathic foot ulcer. Since diabetic foot ulcers are a common occurrence and biopsy of the ulcers is rarely performed, this poses significant challenges in the diagnosis of acral lentiginous melanoma in these patients where diagnosis is often delayed.

We report a case of a diabetic patient who presented at the primary care clinic for a routine follow-up visit with a compliant of an infected ulcer in his right foot which was present for six months. Biopsy confirmed acral lentiginous melanoma, and the patient underwent definitive treatment.

## 2. Case Report

**Figure 1:**
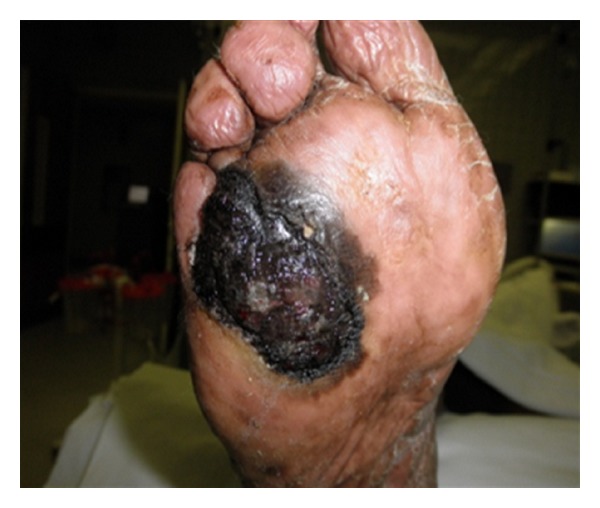
Gross view of the skin lesion on the plantar. The lesion measures 6 × 5 cm with an ulcerated area at the base of fourth and fifth metatarsal involving the interdigital cleft and extending to the forefoot.

**Figure 2:**
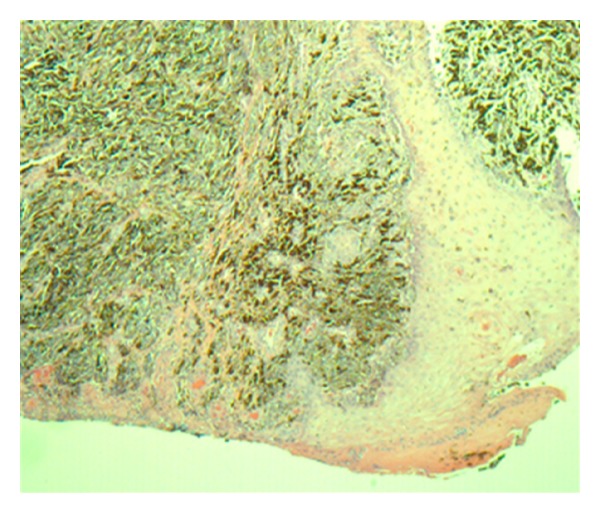
Thickened epidermis ulcerated with many heavily pigmented malignant melanocytes (H and E Stains, 40×).

**Figure 3:**
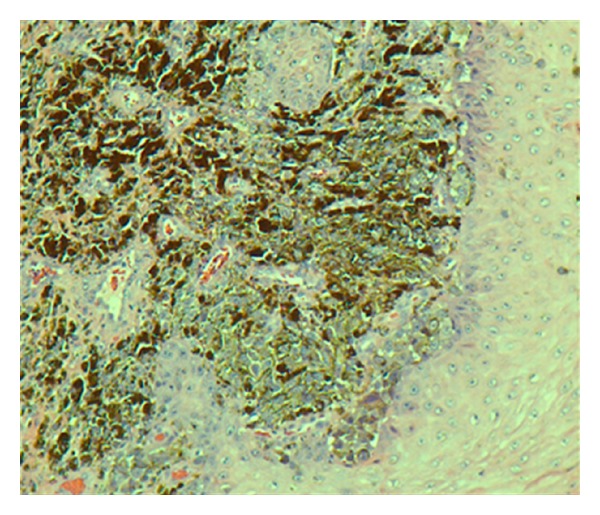
Epidermis and dermis with pigmented malignant melanocytes (H and E stains, 100×).

**Figure 4:**
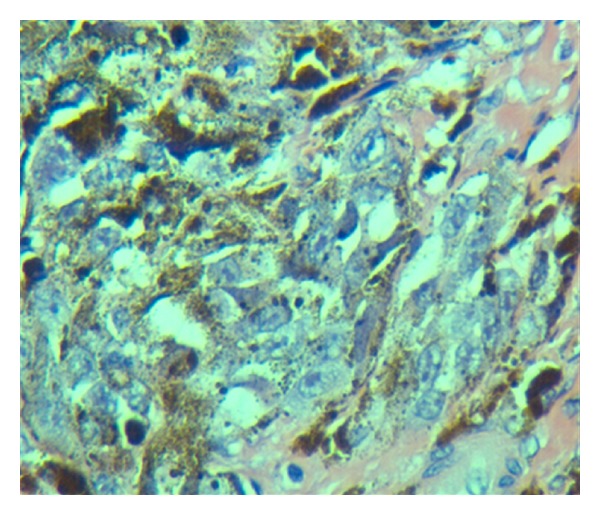
Atypical tumor cells, some pigmented, some not, with large irregular nuclei with large nucleoli. Spindled and round tumor cells. Heavy cytoplasmic melanin pigment (H and E stains, 400×).

**Figure 5:**
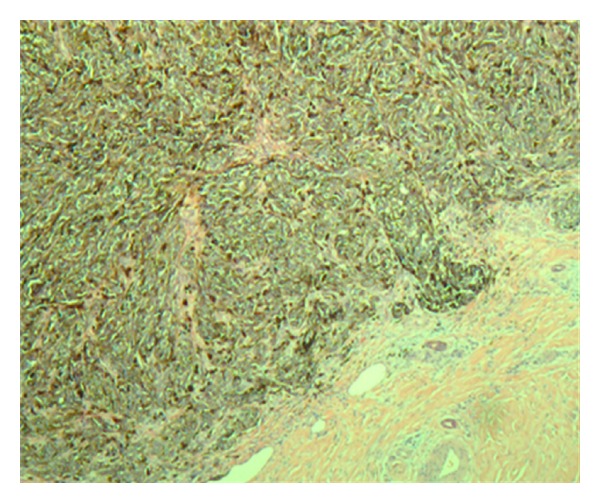
Dermal involvement of tumor cells with uninvolved adjacent deep dermis (H and E stains, 40×).

**Figure 6:**
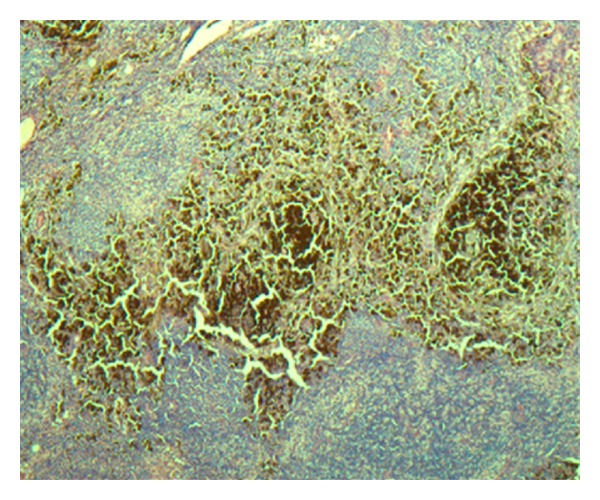
Right groin sentinel lymph node with heavy tumor cell involvement (H and E stains, 40×).

**Figure 7:**
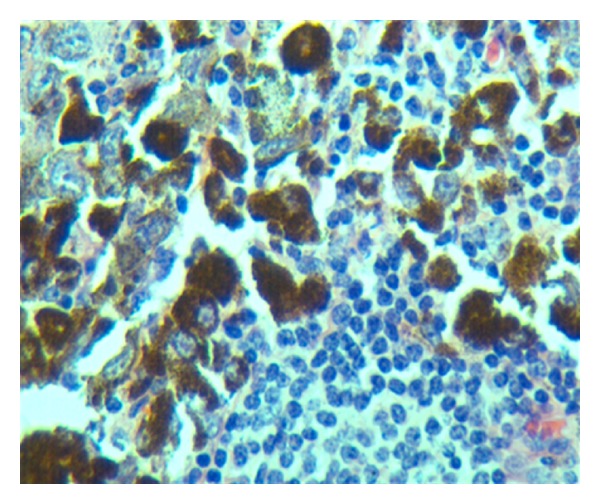
Tumor cells contrasted with benign lymphocytes of lymph node (Hand E stains, 400×).

An 81-year-old African-American male with past medical history of hypertension and diabetes was briefly lost follow up for 9 months. He presented to our primary care clinic for his routine follow-up examination with complaints of a progressively worsening infected right foot ulcer for over six months. Patient also admitted to having pain and foul smelling drainage from the ulcer. Patient denied any tobacco or alcohol use. Physical exam was unremarkable except for an ulcer on the plantar aspect on his right forefoot measuring 6 × 5 cm with an ulcerated area at the base of fourth and fifth metatarsal involving the interdigital cleft and extending to the forefoot (Figure 1). The ulcer was necrotic, black with bleeding and minimal purulent drainage. The patient was started on oral antibiotics with local wound care and surgical consultation requested. Outpatient surgical evaluations revealed a black eschar with punctuate areas of bleeding after the removal of the eschar. The patient was admitted to the hospital for surgical debridement of the presumptive diabetic necrotic ulcer. Laboratory studies including C-reactive protein (CRP) were within normal limits. His hemoglobin A1C was 5.6% indicating excellent control of his diabetes. Radiographic studies of the foot did not show air in the soft tissue, bony involvement, or signs of osteomyelitis. In the operating room under careful examination, the edges of the lesion were irregular with hyperpigmentation highly suggestive of a malignancy. An excision biopsy with 2 mm margin was sent for frozen section and pathology evaluation. Pathologic diagnosis was acral lentiginous melanoma. The tumor had an ulcerated surface that permeated the papillary and reticular dermis without permeation of subcutaneous tissue, suggestive of a Clarke level 4 (Figures 2, 3, 4, and 5). Maximum tumor thickness was 3.5 mm, and mitotic index was <1/mm^2^. A positron emission tomography/computed tomography (PET/CT) was negative for metastatic disease. A definitive procedure was subsequently performed that included en bloc left fourth and fifth toe amputation and wide excision of the previously excised melanoma along with sentinel lymph node biopsy after localizing the node with radioscintigraphy. Patient's sentinel lymph node biopsy was positive for lymph node metastasis (Figures 6 and 7), and his tumor staging, according to the American Joint Committee on Cancer (AJCC) system of TNM (tumor, node, and metastasis) classification, was stage IIIb (T3bN1bM0). Patient refused formal groin and iliac lymph node dissection and was refereed to hematology oncology for chemotherapy treatment with interferon.

## 3. Discussion

The term acral lentiginous melanoma (ALM) was first described by Reed [1] as a subtype of melanoma. It was so named because of its predilection of acral (distal) areas of the body, particularly the palms, soles and the subungual areas, and its distinct radial or “lentiginous” growth phase. ALM represents the rarest of the four subtypes of cutaneous melanoma and yet is the most common variety diagnosed on the foot [2]. Reed described its diagnosis as being based on its histological, intradermal features showing a diffuse proliferation of large atypical melanocytes along the epidermal-dermal junction which is dispersed in a lentiginous pattern with marked acanthosis and elongation of the rete ridges. Research data have demonstrated that melanomas in acral locations account for only 1–7% of all cutaneous melanomas in Caucasians but have been shown to be significantly higher in Asian [3], Chinese [4], Japanese [5], Middle Eastern [6], and African populations [7]. ALM occurs equally across all races, predominantly on an area that seldom receives much sun exposure and suggests that the etiology is different from other subtypes of melanoma. However, Green et al. undertook a case control study of 275 melanomas diagnosed on the soles and palms to investigate risk factors [8]. Interestingly, they found that sun exposure was a significant risk factor in the development of ALM despite their plantar and nail bed location. Furthermore, a high mole count on the soles and elsewhere on the body were associated risk factors (RR = 6.3 95% CI 2.5–15.6). Reinforcing this belief, other studies have demonstrated that increased sun exposure in an individual leads to the development of higher numbers of moles, especially in children [9]. The patient presented here denies any unusual or increased sun exposure. Even though trauma often drew attention to this type of lesion because of the anatomic location, there was no evidence to define it as an etiological factor [10]. Most common clinical features include change in size, color or pattern, and bleeding [11]. Misdiagnosis is a common feature of melanoma on the foot, but ALM in particular has been shown to be more likely misdiagnosed than other subtypes of the disease [12] with a reported rate between 33–37% [13, 14]. One of characteristics of ALM is slow growth. However, the patient we presented here reported the foot ulcer developed for six months only, and he was also under care by a podiatrist 3 months before the ulcer development. One of possibilities for the relatively fast growth of the ALM may point to the diabetes although our patient's diabetes has been well controlled. The study on the melanoma at the Singapore reported higher prevalence of diabetes with ALM patients [15].

## 4. Conclusion

ALM is an uncommon melanoma that can occur in the foot. Early diagnosis and appropriate referral for treatment makes a significant difference in the survival rate and prognosis of the patient. The difficulty in diagnosing it makes it a formidable challenge, especially in this case where it presents as a diabetic foot ulcer. This case once again reiterates the importance of a thorough foot exam in a diabetic patient at each clinic visit.

## Abbreviations

ALM: Acral lentiginous melanoma
US: United States
CRP: C-reactive protein
PET/CT: Positron emission tomography/computed tomography
AJCC: American Joint Committee on Cancer
H and E stains: Hematoxylin and eosin stain.
